# Response of female beetles to LIDAR derived topographic variables in Eastern boreal mixedwood forests (Coleoptera, Carabidae)

**DOI:** 10.3897/zookeys.147.2013

**Published:** 2011-11-16

**Authors:** Timothy T. Work, Benoit St. Onge, J.M. Jacobs

**Affiliations:** 1 Département des Sciences Biologiques, Université du Québec à Montréal; 2 Département de Géographie, Université du Québec à Montréal; 3 Centre de l’étude de la forêt

**Keywords:** boreal carabids, topography, LIDAR, remote sensing, fire skips

## Abstract

Biodiversity monitoring is increasingly being bolstered with high resolution data derived from remote sensing such as LIDAR (Light Detection and Ranging). We derived a series of topographical variables, including slope, azimuth, ground curvature and flow accumulation from LIDAR images and compared these to captures of female carabids in pitfall traps in Eastern boreal mixedwood forests. We developed a series of species-specific logistic models predicting the proportion of females for eight dominant species, including *Agonum retractum*, *Calathus ingratus*, *Platynus decentis*, *Pterostichus adstrictus*, *Pterostichus coracinus*, *Pterostichus pensylvanicus*, *Sphaeroderus nitidicollis* and *Synuchus impunctatus*. We used these models to test three hypotheses related to how the modest topography in boreal forests could influence the availability of microhabitats and possibly potential sites for oviposition and larval development. In general, topographic features such as north facing slopes and high flow accumulation were important predictors of the proportion of females. Models derived from larger scale topography, such as hillsides or small watersheds on the order of ¼-1 ha were better predictors of the proportion of females than were models derived from finer scale topography such as hummocks and small depressions. We conclude that topography likely influences the distribution of carabids based on hydrological mechanisms rather than factors related to temperature. We further suggest based on the scale of responses that these hydrological mechanisms may be linked to the attenuation of past disturbances by wildfire and the propensity of unburned forest patches and fire skips.

## Introduction

Inherent in many biodiversity monitoring programs is a belief that there are landscape units or other easily measurable habitat characteristics that can be consistently related to particular species or assemblages. This perspective is at the heart of coarse filter approaches to biodiversity conservation whereby stand structures and habitat features are often used as surrogates for individual or multiple species (Hunter 1990, Hunter 1991, Franklin et al. 2002). Concordance between habitat features and organisms could expedite biological monitoring by allowing habitats to serve as surrogates for species or identify habitat features that are required for the preservation of biodiversity.

While links between habitat units and organisms will require validation across numerous taxa, many recent efforts have focused on the relation between carabid beetles with landscape units or stand structures (Koivula 2002, Work et al. 2010). Carabid beetles continue to be used in biodiversity monitoring programs primarily because 1) they are relatively easy to sample, 2) they are relatively well described [at least in North America and Europe] and 3) they respond to relatively large-scale changes in habitat that occur at stand-level or larger landscape units [including watersheds or large-scale vegetation classes] (Rykken et al. 1997, Rainio and Niemelä 2003, Work et al. 2008). Carabids have been widely used in the context of forest management to evaluate stand-level changes related to harvesting (Niemelä et al. 1993, Work et al. 2010) or long-term changes in stand succession (Work et al. 2004).

Increasingly, larger-scale classifications such as forest inventory data, ecological landtype phases [ELTPs], and stand maps are being complemented with much higher resolution data on stand structure and underlying topography. One such example is Light Detection and Ranging [LIDAR] which provides detailed digital terrain models [DTMs] and fine scale information related to the topography of surfaces (van Leeuwen and Maarten 2010). This technique is based on computing the precise position of surface returns corresponding to numerous laser pulses emitted from a LIDAR sensor placed onboard a surveying aircraft. Returns occur at the surface of vegetation canopies, but also at ground level, owing to the capacity of the highly collimated laser pulses to travel through dense forests. By interpolating ground returns, DTMs are reconstructed with a vertical accuracy of approximately 30 cm (in conditions where ground is occluded by a dense canopy, (Hodgson and Bresnahan 2004)), and with a resolution as high as 1 meter. For landmanagers these new topographical data are enticing as they provide information at extremely large spatial scales and in some cases permit inferences on landscape-level dynamics (Lefsky et al. 2001, Vepakomma et al. 2008). The high resolution of LIDAR can alternatively be applied to small scales to identify microhabitats or factors that may be important for ‘fine grained’ species such as carabid beetles. LIDAR is indeed being used more frequently in habitat studies (Vierling et al. 2008), particularly for the avifauna (Hinsley et al. 2009). Müller and Brandl (2009) used LIDAR to help explain changes in beetle assemblages [including 62 carabid species] across relatively large elevation gradients (650–1400 m a.s.l.) in Bavaria, Germany. Among the authors’ conclusions was that large gradients in elevation affected beetle communities and LIDAR data explained a large portion of the total variance explained [22%] in a canonical correlation analysis. While this study does demonstrate that techniques such as LIDAR may have a role in habitat mapping for insect communities over large habitat gradients [750 m of elevation over 29.3 km], it does not evaluate the potential of LIDAR in more topographically subtle environments such as the boreal forest where surface features are less pronounced and occur over much smaller scales.

Surface topography could affect carabids in many ways. South-facing slopes in northern climates are often preferentially selected as oviposition sites by a variety of insects. These habitats are often attributed some sort of developmental advantage for immature stages of insects, where south facing oviposition sites may be warmer (Andreson et al. 2001;Weiss et al. 1988). Factors such as surface curvature and flow accumulation govern water flow both above and within soils. Surface curvatures such as hummocks and depressions often provide essential microgradients for less motile organisms such as bryophytes and plants (Weltzin et al. 2001, Flinn 2007). Areas with seepage, where the flow of water both above and below ground, is thought to be important, have been casually implicated in biodiversity hotspots by some forest managers (T. Vinge pers. comm.). For litter and soil dwelling arthropods, particularly immature stages, soil moisture is an important factor. For temperate species who develop as larvae in summer [often spring-summer breeders as adults], microsites such as depressions or other sites that accumulate water may provide some protection against desiccation (Luff 1993), particularly in areas where forest cover may be limited or absent such as gaps, defoliated stands or cuts. For temperate species who overwinter as larvae [often autumn breeders as adults], these same sites may be detrimental if winter snowmelts cause flooding and mortality of larvae (Luff 1993).

Here we have related changes in the proportion of females for eight dominant carabid species in boreal forests to topographic variables derived from LIDAR images. We concentrated our analysis on four primary topographic elements; slope, azimuth, curvature and elevation, and one secondary element; flow accumulation derived from a high precision, LIDAR digital elevation map in order to examine three hypotheses related to microhabitat preference of female carabids.

We propose three working hypotheses which relate surface topography to the proportion of females with the underlying assumption that this proportion reflects preferred oviposition sites and initial larval habitats (Lövei and Sunderland 1996). Our first hypothesis predicts a greater proportion of females on south facing slopes where insulate radiation and warmer conditions would speed larval development. Our second hypothesis is related to hydrological effects of topography and resistance to desiccation. We expect that species commonly associated with closed canopy forests will have higher proportions of females in proximity to hydrological features such as high flow accumulation or surface concavities, particularly small scale features such as depressions or concavities. These are sites where risk of larval desiccation would be minimized. Our third hypothesis relates combined influences of topography on the frequency, extent and severity of wildfire. Under this hypothesis, we predict a higher proportion of females on northern aspects with hydrological elements where the impacts of fire may be limited and less frequent and where unique habitats may be more frequent. While any hypothesis related to fire is strongly related to hypotheses based on moisture, this third hypothesis would likely be specific to larger scale topographic features that correspond to the size of fire skips.

## Methods

### Beetle trapping

All carabids were collected using pitfall traps at the SAFE research site in the Lac Duparquet Research and Teaching Forest in northwestern Québec (Brais et al. 2004). Beetles were collected from replicated stands within three forest cohorts. These cohorts were characterized as 1) deciduous cohort dominated by trembling aspen originating from wildfire in 1923, 2) mixed cohort were trembling aspen and balsam fir are present in the overstory originating from wildfire in 1910 and 3) post-budworm/birch cohort where nearly all large balsam fir were killed in the most recent spruce budworm outbreak and which originated from wildfire in 1760. These cohorts represent a gradient in succession/natural disturbance common in the Eastern boreal forest. All beetles were sampled using pitfall traps /8.5 cm diameter/ between 2004 and 2006 as part of a larger project related to coarse-filter management and carabid biodiversity /O’Connor in preparation/. Pitfall traps were collected approximately every three weeks between between 8 May and 26 August in 2004 and between 28 May and 8 September in 2005 from aspen stands. Samples were collected from mixed and balsam fir stands between, 28 May and 8 September in 2005 and 6 May to 23 September 2006. In all years, pitfall traps were deployed when snow cover was still abundant in forest stands. In each stand, 10 pitfall traps were deployed as paired traps, spaced 10 m apart centered on permanent sampling plots. These permanent sampling plots were randomly positioned within each stand (Brais et al. 2004). For all analyses, we used pooled catches from the paired traps at each of the 44 permanent sampling plots as the unit of analysis. We limited our analysis to 8 abundant species; *Agonum retractum* LeConte, *Platynus decentis* (Say), *Calthus ingratus* Dejean, *Pterostichus adstrictus* Eschscholtz, *Pterostichus coracinus* (Newman), *Pterostichus pensylvanicus* LeConte, *Sphaeroderus nitidicollis* Guérin-Méneville and *Synuchus impunctatus* (Say). These 8 species represented 91% of the total carabid abundance. While exact breeding times varies by species, each of these species could be characterized as a spring breeder (Larochelle and Larivière 2003).

### Deriving topographic variables

LIDAR data was acquired in full-leaf conditions on 14-16 August 2003 using an Optech ALTM 2050 sensor flown at 1 000 m above ground level. The sensor recorded the first and last returns of each laser pulse emitted at 50 000 Hz. The last returns were classified into ground and non-ground categories by the data provider using the Terrascan application from Terrasolid Ltd (Helsinki, Finland). The last returns classified as ground were interpolated and gridded at resolutions of 4, 8, 16 and 32 m to create multi-scale DTMs. Topographic variables were derived from these LIDAR DTMs and included local slope, azimuth, curvature and flow accumulation using ArcGIS 9.3 (ESRI, Redlands CA). Slopes were defined as a percentage where 100% would be equivalent to 45°. Azimuths were translated to ‘northing’ where values range from 0-180° with 0° reflecting N and 180° reflecting S, thus eliminating the E-W axis from azimuth. Curvature is defined from second-order derivatives from a 4^th^-order polynomial surface based on elevation. Positive values represent hummocks where as negative values represent depressions. Flow accumulation (FAC) corresponds to the number of grid cells that are connected to a target cell through monotonic increases in elevation. We recognize that this measure cannot be interpreted in the strict sense of fluids passing over impermeable surfaces, but nevertheless we feel that this provides some insight into runoff following heavy precipitation events and soil hydrology. Each of these variables was derived by placing a 3 × 3 cell grid centered on pitfall trap locations. We derived each variable at four spatial scales corresponding to the DTM’s grid cell sizes of 4, 8, 16 and 32 m. Thus topographic variables reflect increasingly larger surface areas or neighborhoods [144, 576, 2034, 9026 m^2^]. By changing the scale of inference on topographic variables we feel that we capture a range of perspectives reflecting ‘microsites’ to ‘seepage’.

### Relating carabids to topographic parameters

The proportion of females captured for each species were related to the five topographic parameters at each scale using logistic regression. Prior to this analysis we evaluated a series of logistic-mixed models, where individual forest cohorts were treated as random effects in the model. In each case, these random variations among cohorts were extremely small, thus we simplified our approach using simple logistic models.

For each logistic model, the five topographic variables were centered to a mean of 0. We used AIC to select models that best reflected changes in the proportion of females to four scales of LIDAR derived topographic variables. Once an optimal scale was selected, the full model containing all five topographic variables was compared to four reduced models using AICc. These reduced models were chosen to reflect hypotheses related to female responses to temperature, moisture and elevation. The reduced models were 1) slope and azimuth, 2) slope, azimuth and elevation, 3) curvature and FAC, and 4) curvature, FAC and elevation. For each model, we corrected for model dispersion using the sum of squared pearson`s residuals divided by residual degrees of freedom. Models were constructed using the glm function in R 2.11.1 (R Development Core Team 2010).

## Results

### Topography

The mean and standard deviation of slope and curvature decreased with increasing scales (Table 1). Thus at larger spatial scales topography became flatter and more planar. In contrast, aspect, elevation and FAC were largely consistent across scales. Together these variables typify a boreal landscape with modest changes in slopes which are distributed in all directions and where small, ‘fine-grained’ topographic features [such as small hummocks and depressions] are the norm (Table 2). Differences in elevation across all scales were equally modest, ranging from 38-43 m.

**Table 1.** Means and standard deviations of topographic parameters derived at four spatial scales from LIDAR.

**Parameter**	**4m**		**8m**		**16m**		**32m**	
	**Mean**	**SD**	**Mean**	**SD**	**Mean**	**SD**	**Mean**	**SD**
Azimuth	83.14	59.724	78.45	62.7	84.63	60.746	61.46	58.978
Slope	9.87	6.399	7.39	3.47	4.11	2.605	2.18	2.006
Curvature	-0.07	0.786	0.02	0.267	0	0.041	0	0.021
FAC	12.46	34.021	10.1	11.079	17.71	42.16	15.49	19.46
Elevation	249.7	11.443	249.76	11.41	248.94	12.275	251.33	15.569

**Table 2.** Absolute range of topographic parameters derived at four spatial scales from LIDAR.

**Parameter**	**4m**	**8m**	**16m**	**32m**
Azimuth	173.60	176.86	167.37	174.16
Slope	27.39	17.70	9.39	6.71
Curvature	3.94	1.25	0.20	0.14
FAC	216.00	49.00	228.00	79.00
Elevation	38.21	38.52	38.85	43.17

### Response of female carabids to scale and topography

With the exception of *Agonum retractum*, the proportion of females was related to topographic variables derived at 16 and 32 m scales (Table 3). With the exception of *Platynus decentis*, the proportion of females observed was best explained by reduced models which had consistently lower AICc values than the initial full model.

**Table 3.** AIC values for full logistic models based on azimuth, slope, curvature, flow accumulation and elevation.

Species	4m	8m	16m	32m
*Agonum retractum*	148.651	151.444	151.012	155.601
*Calathus ingratus*	72.711	73.287	70.220	73.112
*Platynus decentis*	126.694	142.549	138.993	124.737
*Pterostichus adstrictus*	178.912	178.274	181.027	175.792
*Pterostichus coracinus*	130.115	130.577	122.559	129.707
*Pterostichus pensylvanicus*	225.792	224.805	223.546	219.138
*Sphaeroderus nitidicollis*	133.667	134.729	131.134	137.884
*Synuchus impunctatus*	160.347	160.566	159.233	159.532

**Figure 1.**
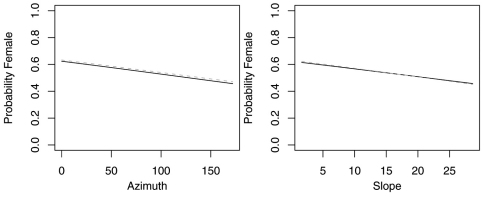
Probability of collecting female *Agonum retractum* with changes in azimuth and slope when additional variables are held constant at their respective means. Dashed lines represent model parameters derived from full logistic model. Solid lines represent model parameters of reduced logistic model including only azimuth, slope and intercept.

**Figure 2.**
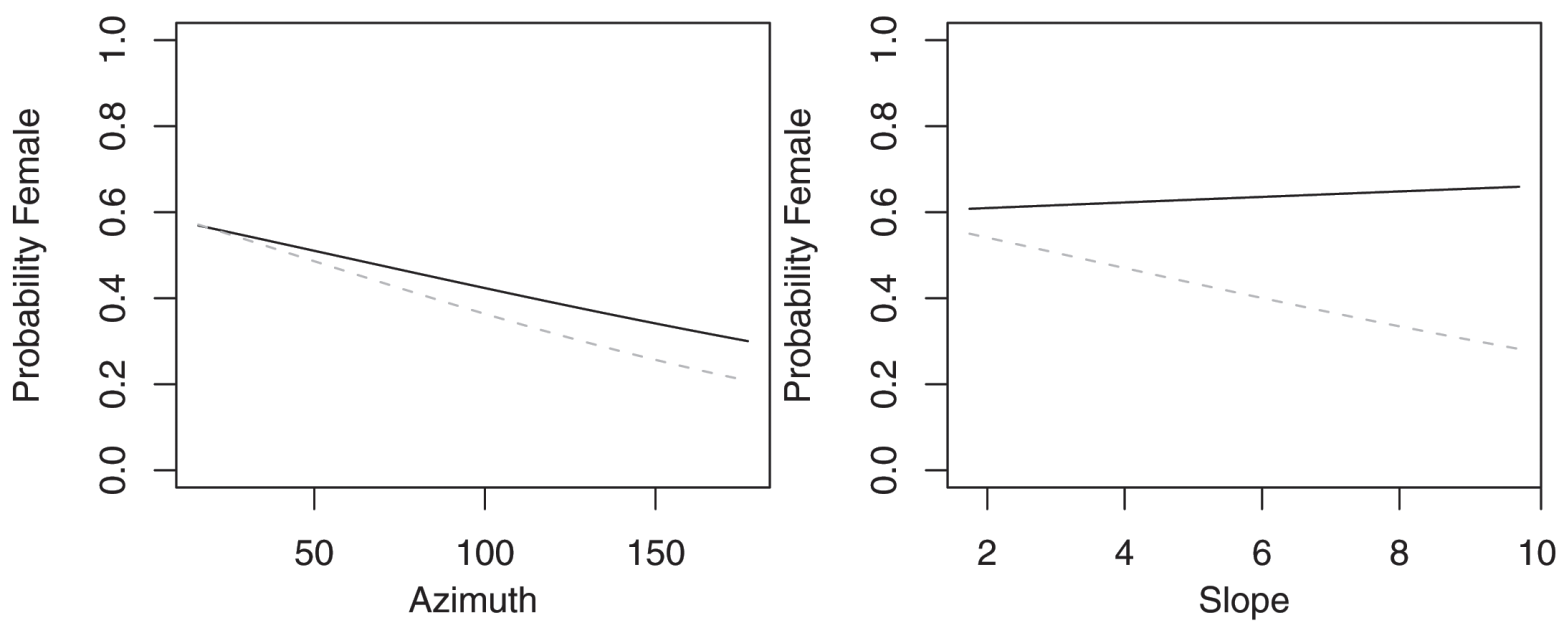
Probability of collecting female *Calathus ingratus* with changes in azimuth and slope when additional variables are held constant at their respective means. Dashed lines represent model parameters derived from full logistic model. Solid lines represent model parameters of reduced logistic model including only azimuth, slope and intercept.

**Figure 3.**
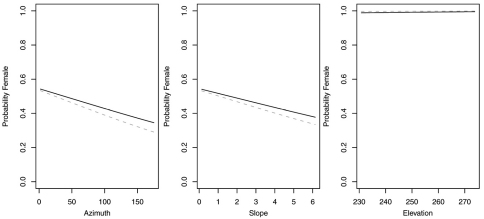
Probability of collecting female *Pterostichus adstrictus* with changes in azimuth, slope and elevation when additional variables are held constant at their respective means. Dashed lines represent model parameters derived from full logistic model. Solid lines represent model parameters of reduced logistic model including only azimuth, slope, elevation and intercept. Regression slopes for elevation appear near zero as total range of elevation is relatively small.

**Figure 4.**
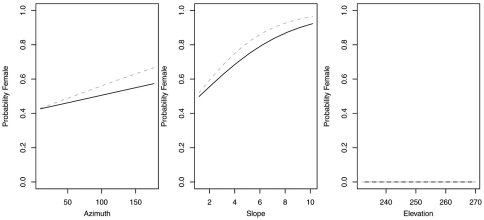
Probability of collecting female *Pterostichus coracinus* with changes in azimuth, slope and elevation when additional variables are held constant at their respective means. Dashed lines represent model parameters derived from full logistic model. Solid lines represent model parameters of reduced logistic model including only azimuth, slope, elevation and intercept. Regression slopes for elevation appear near zero as total range of elevation is relatively small.

The reduced model with only azimuth and slope was the best model for *Agonum retractum* (Figure 1) and *Calathus ingratus* (Figure 2), although in both cases azimuth was the only significant predictor (Table 4). In both these cases, the proportion of females increased on more northern azimuths. The odds of finding *Agonum retractum* and *Calathus ingratus* females on northern azimuths were 1.97 (95% CI=1.144 to 3.398) and 3.083 (95% CI= 1.139 to 8.340) times greater, respectively than on southern azimuths. Reduced models for *Pterostichus adstrictus* and *Pterostichus coracinus* also included azimuth and slope but were expanded to include elevation based on AICc comparisons (Table 5). Elevation was a significant predictor for both species while proportion of *Platynus adstrictus* females was larger on northern azimuths (Figure 3) and proportion of *Pterostichus coracinus* females increased with slope (Figure 4). The odds of finding *Platynus adstrictus* females were 2.249 (95% CI= 1.034 to 4.892) times greater on northern azimuths than southern azimuths and 2.228 (95% CI= 1.034 to 4.798) times greater at its maximum elevation than its minimum elevation. The odds of finding *Pterostichus coracinus* females were 6.394 (95% CI= 1.956 to 20.897) times greater on its maximum slopes than its minimum slopes and 10.539 (95% CI= 3.290 to 33.759) times greater at its minimum elevation than its maximum elevation.

**Table 4.** Reduced logistic models for *Agonum retractum* [4m scale] and *Calathus ingratus* [16m scale] ^1^.

	**Estimate**	**Std. Error**	**z value**	**Pr(>|z|)**
*Agonum retractum*				
(Intercept)	0.5059	0.09270	5.45800	0.00000
Azimuth	-0.0039	0.00161	-2.44700	0.01440
Slope	-0.0236	0.01562	-1.51200	0.13050
*Calthus ingratus*				
(Intercept)	0.3904	0.14514	2.69000	0.00714
Azimuth	-0.0070	0.00315	-2.21700	0.02662
Slope	0.0276	0.06661	0.41500	0.67834

^1^ P-values were corrected with 0.925518 and 0.585495, respectively as dispersion parameters

**Table 5.** Reduced logistic models for *Pterostichus adstrictus* [32m scale] and *Pterostichus coracinus* [16m scale] ^1^.

	**Estimate**	**Std. Error**	**z value**	**Pr(>|z|)**
*Pterostichus adstrictus*				
(Intercept)	0.1793	0.10270	1.74600	0.08080
Azimuth	-0.0047	0.00229	-2.04400	0.04090
Slope	-0.1107	0.07034	-1.57300	0.11560
Elevation	0.0186	0.00907	2.04600	0.04080
*Pterostichus coracinus*				
(Intercept)	-0.3303	0.12082	-2.73400	0.00626
Azimuth	0.0035	0.00239	1.48000	0.13881
Slope	0.2766	0.09006	3.07100	0.00214
Elevation	-0.0546	0.01376	-3.96500	0.00007

^1^ P-values were corrected with 1.293246 and 0.889476, respectively as dispersion parameters.

**Figure 6.**
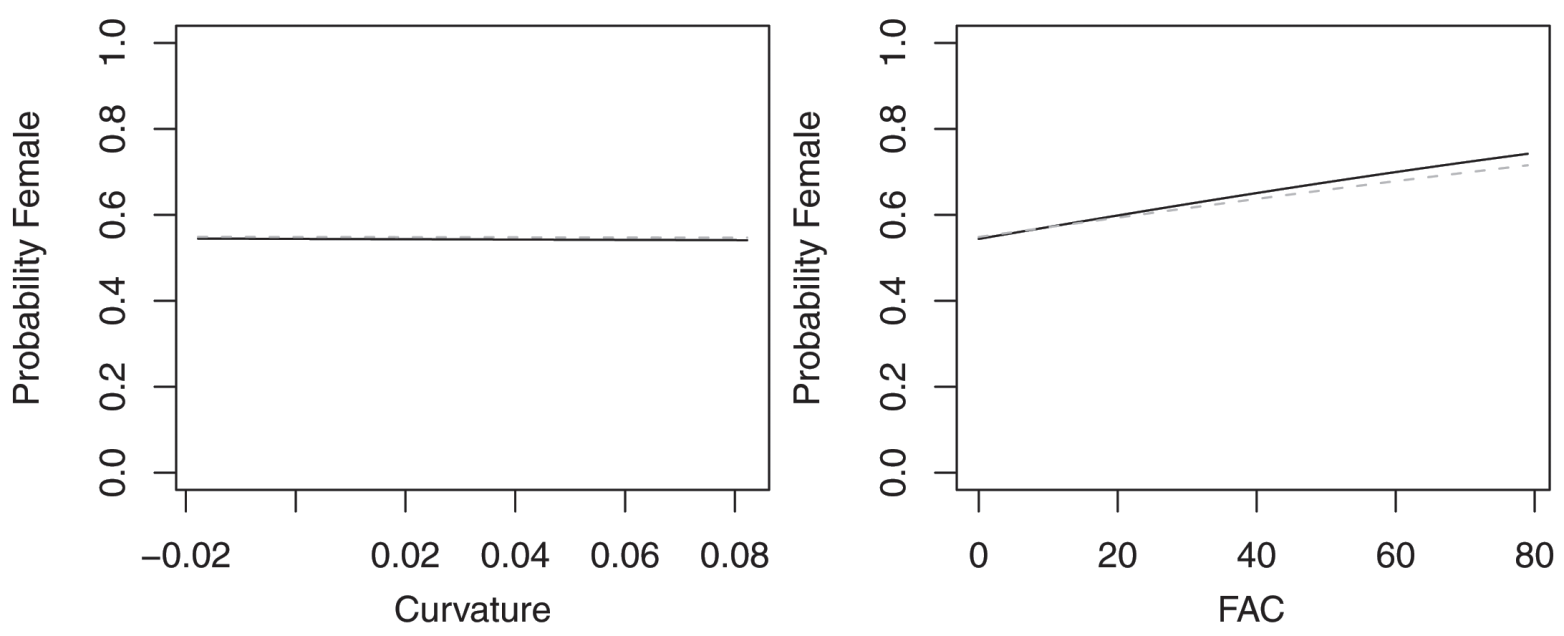
Probability of collecting female *Pterostichus pensylvanicus* with changes in azimuth and slope when additional variables are held constant at their respective means. Dashed lines represent model parameters derived from full logistic model. Solid lines represent model parameters of reduced logistic model including only curvature, FAC and intercept.

**Figure 7.**
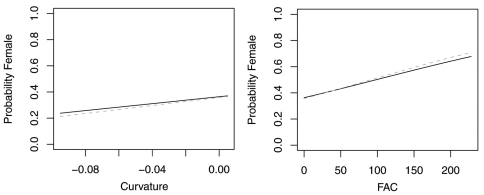
Probability of collecting female *Sphaeroderus nitidicollis* with changes in azimuth and slope when additional variables are held constant at their respective means. Dashed lines represent model parameters derived from full logistic model. Solid lines represent model parameters of reduced logistic model including only curvature, FAC and intercept.

**Figure 8.**
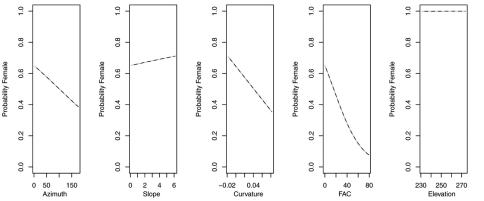
Probability of collecting female *Platynus decentis* with changes in azimuth and slope when additional variables are held constant at their respective means. Both dashed and solid lines represent model parameters derived from full logistic model. Regression slopes for elevation appear near zero as total range of elevation is relatively small.

In contrast, reduced models for *Synuchus impunctatus*, *Sphaeroderus nitidicollis* and *Pterostichus pensylvanicus* included only curvature and FAC, rather than slope, aspect or elevation (Table 6). In the reduced model for *Synuchus impunctatus*, neither curvature nor FAC were significant (Figure 5). Proportion of females for *Pterostichus pensylvanicus* increased with increasing FAC (Figure 6). The odds of finding female *Pterostichus pensylvanicus* were 2.411 (95% CI= 1.126 to 5.164) times greater in areas at its maximum FAC than areas with low FAC. Proportion of females of *Sphaeroderus nitidicollis* increased with both increasing FAC and curvature (Figure 7). The odds of finding female *Sphaeroderus nitidicollis* were 3.693 (95% CI =1.190 to 11.465) greater at its maximum FAC than on its minimum FAC and 3.521 (95% CI=1.493 to 8.300) times greater in areas of its maximum curvature than minimum curvature. *Platynus decentis* was the only species where the full topographical model best explained changes in the proportion of females based on model comparisons (Table 7), however, like *Sphaeroderus nitidicollis* only FAC and curvature were significant predictors of changes in the proportion of females (Figure 8). However, unlike *Sphaeroderus nitidicollis*, *Platynus decentis* was negatively associated with both FAC and curvature. The odds of finding *Platynus decentis* were 22.000 (95% CI = 3.233 to 149.714) times greater on sites with minimum FAC than on sites with maximum FAC. The odds of finding *Platynus decentis* were 8.499225 (95% CI = 1.736651 to 41.59547) greater at areas with minimum curvature than areas with maximum curvature.

**Table 6.** Reduced logistic models for *Pterostichus pensylvanicus* [32 m scale], *Sphaeroderus nitidicollis* and *Synuchus impunctatus* [both at 16m scale]^1^.

	**Estimate**	**Std. Error**	**z value**	**Pr(>|z|)**
*Pterostichus pensylvanicus*				
(Intercept)	0.1767	0.07114	2.48400	0.01300
Curvature	-0.1532	1.93062	-0.07900	0.93680
FAC	0.0111	0.00492	2.26500	0.02350
*Sphaeroderus nitidicollis*				
(Intercept)	-0.5619	0.10347	-5.43100	0.00000
Curvature	6.3393	2.87685	2.20400	0.02760
FAC	0.0057	0.00254	2.26100	0.02380
*Synuchus impunctatus*				
(Intercept)	0.6328	0.12900	4.90600	0.00000
Curvature	3.9788	3.81754	1.04200	0.29700
FAC	0.0001	0.00353	0.03000	0.97600

^1^P-values were corrected with 1.487152, 0.8426052 and 1.456111, respectively as dispersion parameters

**Figure:**
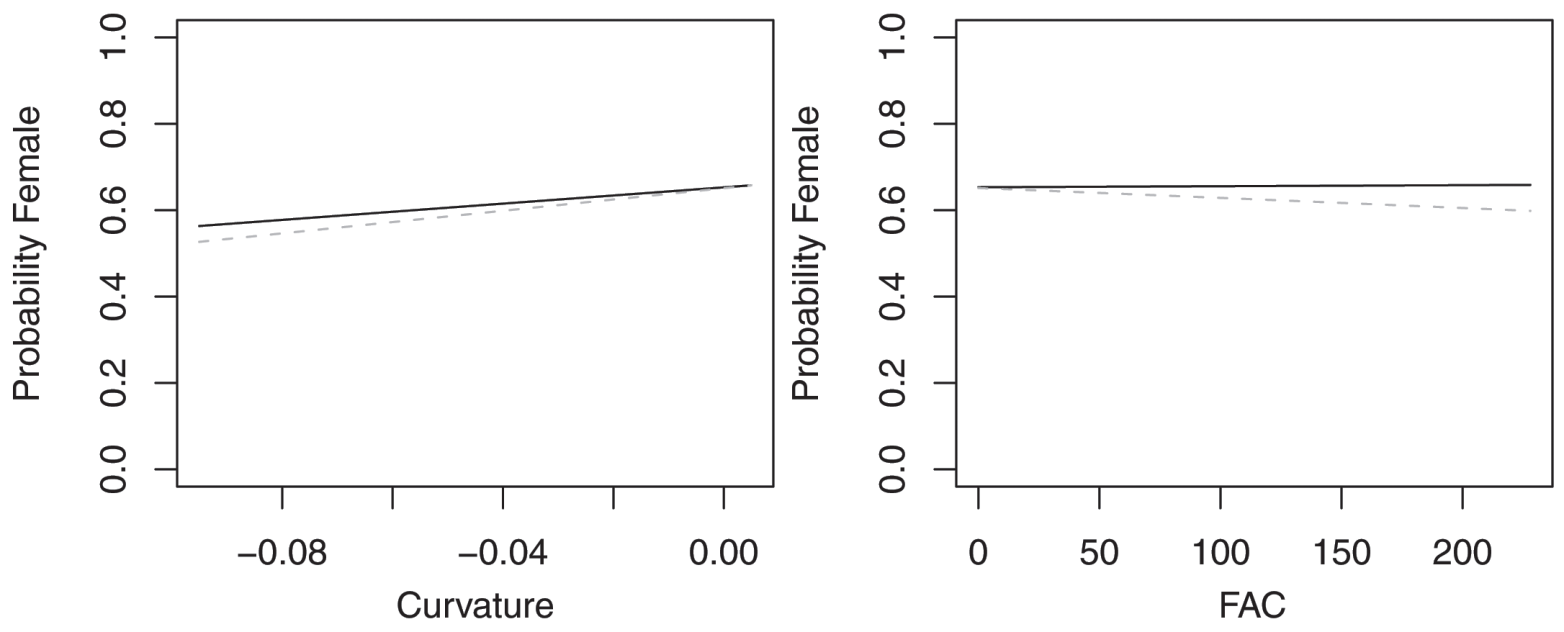
**Figure 5.** Probability of collecting female *Synuchus impunctatus* with changes in azimuth and slope when additional variables are held constant at their respective means. Dashed lines represent model parameters derived from full logistic model. Solid lines represent model parameters of reduced logistic model including only curvature, FAC and intercept.

**Table 7.** Reduced logistic models for *Platynus decentis* [32 m scale]^1^.

	**Estimate**	**Std. Error**	**z value**	**Pr(>|z|)**
(Intercept)	0.6225	0.13125	4.74300	0.00000
Azimuth	-0.0062	0.00343	-1.79600	0.07252
Slope	0.0455	0.18603	0.24500	0.80672
Curvature	-14.8768	5.63250	-2.64100	0.00826
FAC	-0.0391	0.01239	-3.15900	0.00158
Elevation	0.0287	0.02251	1.27300	0.20289

^1^P-values were corrected with 1.101403 as a dispersion parameter

## Discussion

Our data suggest that females for all but one species are responding to topographic features on the order of 1/4-1 ha rather than highly localized microsites [i.e.<1/20 ha]. While it is tempting to further relate body size as a surrogate for dispersal ability under a hypothesis that ‘large species operate at larger scales’, our data does not support this hypothesis. While *Agonum retractum*, the smallest species used in our analysis, responded to the smallest scale of topographic variables, all other species were associated with larger scales [16-32 m]. To rigorously test this hypothesis, more species should be included along with a larger range of spatial scales.

Neither does our data correspond directly with either of our hypotheses related to development time or small scale hydrology. Species that oviposit preferentially with slope and aspect in order to speed or synchronize larval development are often phytophagous and strongly linked to plant phenology (Andresen et al. 2001; Weiss et al. 1988). Initially, we reasoned that predacious species would profit from a similar advantage if they were implicated in size structured food webs and rapid passage to adult stages conferred either reduced mortality or access to larger and presumably more profitable prey. The fact that none of carabids we analyzed were positively associated with southern slopes discounts this hypothesis. The fact that no species responded to smaller scale features such as pits or mounds, suggests that these smaller features are simply part of the natural heterogeneity encountered by these species. Rather we prefer to concentrate on the landscape elements present at this scale and its relation to fire history to explain the differences in distribution of females.

In our study site, ¼-1 ha corresponded to topographic features on the order of small hillsides or catchments rather than hummocks or depressions related to uprooted trees, large rocks or buried logs. In the eastern boreal forest, modest differences in topography are nested within a landscape where heterogeneity is maintained by large-scale, stand replacing fire. This fire regime results in a skewed distribution where there are numerous small fires [on the order of <1-10 ha] and relatively few very large fires (Bergeron et al. 2004; Cumming 2001). In certain cases, Bergeron and Brisson (1990) have shown that under mesic conditions fires can be less than 1 ha and of moderate or low severity, while fire size and intensity increased in more xeric conditions. For these smaller patches of forest, topographic features such as high FAC or more northern, and presumably moister, slopes on the order of 1/4-1 ha comprise a relatively large proportion of the potential affected area and could conceivably minimize fire severity and promote and maintain fire skips. Similarly, Cyr et al. (2005) have suggested that distance to firebreaks and water courses could decrease [although not negate] risk of fire. While much of the discussion related to topography and fire has concentrated on spatial scales larger than those examined in this study, aspect seems to be an important feature at these large scales where northern facing slopes reduce risk of fire by a factor of 3 as compared to southwestern slopes (Cyr et al. 2007). Nevertherless, small fire skips (<1 ha), have been shown to be important features for certain carabid species such as *Pterostichus empetricola* (Dejean) in the western boreal (Gandhi 2006). It is conceivable that females have developed a preference or at least are more prevalent on northern aspects as oviposition sites as a means of avoiding mortality from these periodic disturbances. Alternatively, increased prevalence of females could also be considered the legacy of environmental filtering by these periodic disturbances resulting in the present distribution of females across a range of topography. We are highly skeptical that female carabids are capable of evaluating long-term disturbance dynamics, rather we suspect that more proximal factors related to moisture and aspect are used to evaluate viable oviposition sites, but it is possible that longer-term dynamics related to natural disturbances have resulted in the present distribution of carabid females. Presently we are incapable of evaluating whether the distribution observed in this study [taken over 3 years] is consistent over longer time scales. If so, this would provide more circumstantial evidence that female carabids are responding to habitat conditions which promote fire skips.

The only species associated with small scale topography, *Agonum retractum* was positively associated with northern azimuths. Providing scale specific interpretations of azimuth, as compared to other topographic variables, is difficult as azimuth varies little over different spatial scales. Thus physical explanations related to northern azimuths such as retention of snow and thus higher humidity could also be applied to smaller scales such as the northern sides of hummocks.

Alternatively, the smaller scale responses of *Agonum retractum* may be a methodological artifact related to elevation errors in the LIDAR image. Elevational errors are caused in part by variations of the vertical accuracy of LIDAR under forest canopies. These errors are influenced by ground return density and by the ground/non-ground classification success. Derivatives such as slope, aspect and FAC are quite sensitive to small scale elevational errors and may be relatively noisy at high resolution. This noise would in turn greatly weaken statistical relationships to observed insect abundance. At larger scales, minute LIDAR errors cancel out leading to increased robustness of derivatives.

The fact that we are able to detect changes in the proportion of females in response to very small changes in topography demonstrates the potential of high-resolution remote sensing techniques such as LIDAR, which is currently the only 3D remote sensing instrument capable of creating accurate and high resolution topographical maps, a capacity that opens up numerous research paths. In the recent past, LIDAR-based habitats studies have focused on flora, or larger animals such as birds or mammals. We have additionally demonstrated that LIDAR DTMs can be useful for studying the habitat preferences of insects at different scales, even for a single insect family in a landscape with very mild topography.

## Conclusions

We believe that advances in remote sensing such as LIDAR will likely continue to complement biodiversity assessments rather than replace actual biological inventories as a stand-alone surrogate. While it is initially seductive to mine high resolution remote sensing data for correlations with assemblages, this seems more of a preliminary exercise in calibration of the technique than an end goal. The utility of LIDAR will be borne out when the force of this data is realized through mechanistic hypotheses related to habitat requirements of plants and animals. We have shown that topographic features such as north facing slopes and sites with high FAC are more likely to harbor female carabids in eastern boreal landscapes where topography is modest, although the current imprecision of these models clearly limits predictive applications of these models. We further demonstrate that 7 of the 8 species analyzed respond to topography consistent with the scale of fire skips and suggest that topography may be affecting the distribution of females by maintaining more mesic habitats and attenuating wildfire. This in itself is a preliminary hypothesis which assumes a strong link between distribution of females, oviposition choice and larval survivorship. However we hope that using LIDAR to identify preferred microhabitats for females will lead us to a better understanding of preferred habitats of larvae which we feel has been largely overlooked in discussions of carabids as bioindicators or in discussions of refining coarse filter approaches to conservation.
